# In search of traditional bio-ecological knowledge useful for fisheries co-management: the case of jaraquis *Semaprochilodus *spp. (Characiformes, Prochilodontidae) in Central Amazon, Brazil

**DOI:** 10.1186/1746-4269-6-15

**Published:** 2010-06-03

**Authors:** Vandick S Batista, Liane G Lima

**Affiliations:** 1 Universidade Federal de Alagoas/UFAL, Instituto de Ciências Biológicas e da Saúde/ICBS-LABMAR, Av. Aristeu de Andrade, 452, Farol, CEP: 57051-090, Maceió, Alagoas, Brazil; 2 Universidade Federal do Amazonas/UFAM. Av. Gen. Rodrigo Octávio Jordão Ramos, 3000, Mini-Campus. Bairro Coroado I. CEP 69077-000. Manaus, Amazonas, Brazil

## Abstract

The jaraquis (*Semaprochilodus spp.*) are the most abundant group in the fishing landing in Manaus. However, just command and control management strategies have been used by the fishery governmental agency in the region without the power to enforce centralized decisions. The fishermen and their culture represent a source of information on dynamics of the resources and aquatic environments, fundamental in making possible the co-management of the fishing resources. The present study aims to contribute to management through identification of common information available in scientific and traditional knowledge about the jaraquis' bio-ecology. There were 57 semi-structured interviews recorded with fishermen of Manaus and rural areas of Manacapuru in 2002 concerning biological and ecological aspects. Similarity was observed between scientific and traditional knowledge in the following items: size of first sexual maturation, spawning type, parental care, trophic relationships and migratory behavior, as well as in some aspects of the mortality and growth of the species. However, there was less ethnoicthyological information on fecundity and the determination of the age and growth of adult fish. Common information would be used preferably by agencies to start an effective and technical dialogue with commercial and riverine fishermen to design management plans in a decentralized strategy.

## Background

Fishing is a traditional activity in the Amazon region performed by Indians and local people for direct feeding, generally called subsistence fishery. Commercial activity became more intensive from the end of the decade of the 1960's with the introduction of the monofilament nylon twine, styrofoam boxes in the coating of thermal boxes for fish caught and of diesel motors in boats [1]. Professional fisherman developed from being subsistence fishermen and owing to of such technological improvements became full time fishermen fishing in distant waters to sell to freezing houses and fish markets of urban centers [2,3].

This commercial fishing initially benefitted from the existing tambaqui (*Colossoma macropomum*) fishing resource, which was of greater market value and availability and which attained the yield landed in Manaus of 14 thousand tons per year until the end of the 1970's [4,5]. However, this resource reduced progressively during the 1980s. At the same time the jaraquis (*Semaprochilodus *spp.) reached the maximum yield landed [4,6].

The genera Semaprochilodus belongs to the family Prochilodontidae, with two species in Central Amazon: the jaraqui-escama-fina (*S. taeniurus*) and the jaraqui-escama-grossa (*S. insignis*). Both species were among the most abundant fish landed in Manaus between 2001 and 2003 [7]. In spite of the importance of this and also of the suggestion of overfishing [8-10], it has not been included in the closed fishing season determined yearly by the national organ of natural resources management (IBAMA). On the other hand, it is affected directly by two legal norms: one aims to protect reproductive areas and other to protect young fish (minimum size of catch of 20 cm in total length - TL). However, considering that the application of such norms is occasional and inefficient, it is necessary that a viable management procedure be developed allowing the sustainability of the Amazon fishing resources.

These scientific knowledge has been used traditionally to evaluate fish stocks but has not been easily accepted by fishermen, particularly artisanal fishermen or even government managers. In the last decade many researchers indicated fishermen culture as important source of information for simple evaluation of natural stocks (e.g. [11]). Fishermen culture also can take an important place in the development and effectiveness of fisheries management [12], particularly when command and control strategies cannot be used. In Brazil, Posey, Diegues, Marques and Begossi pioneer researches (e.g. [13-16]) gave the main references to the actual principles and methods used.

In this context, the use of traditional ecological knowledge associated to scientific knowledge should occupy a fundamental place. Active participation of users of resources in the management conception and actions has been frequently discussed [17,18]. However, classical fisheries assessment knowledge is not usually understood by stakeholders and fishermen representatives, whose knowledge is fundamental to the efficiency and credibility of any activity to be performed. Traditional fisheries assessment knowledge has been registered in the region on a few occasions (e.g. [19,20]). As a consequence, the relationship of this knowledge with the scientific knowledge used by the government agencies has rarely been established.

The present study aims to evaluate the potential of traditional bio-ecological knowledge of subsistence and commercial fishermen about the jaraquis in order to contribute to the fisheries management. The results of the assessment were related to similar ones already available in scientific literature evaluating the association of themes to information. Results of fisherman types were also compared, allowing an initial evaluation about the usefulness of the respective sources as a base of information for co-management.

## Methods

The scientific data concerning the biology and ecology of *Semaprochilodus taeniurus *and *S. insignis *were obtained by means of bibliographical revision of published scientific literature, and also by compiling theses, dissertations, monographs and books specialized in the subject. The collection of primary data was made in the fish landing raft in Manaus and in rural communities in a rural zone of the municipal district of Manacapuru, Amazonas, between the latitudes 3° 30' S at 3° 40' S and longitudes 61° 00' W at 60° 45' W.

Structured interviews were done with 57 fishermen between July and December 2002. Thirty one interviewees were professional fishermen from Manaus, called city-based fishermen (following [2]), and 26 with the riverine fishermen of the rural areas of Manacapuru. The interviews were done individually with each fisherman, using a questionnaire with questions concerning personal characteristics, experience, and social relationships in fishing and bio-ecological knowledge of reproduction, feeding, predatory behavior, growth, migration, mortality and recruitment related to the species in question. The research was done in accordance with the ethical and legal obligations of the institution and the country in which the research took place at the time of the research.

The fishermen were randomly selected for interviews of between 45 and 60 minutes. Whole interviews were registered in writing, and soon after inserted in a database for analysis.

Dynamic tables, histograms and associated averages and confidence intervals were used to test treatments. The statistical test Z was used in the comparison of proportions with the null hypothesis where the information was the same [21]. The assumption of normality was evaluated by the Kolmogorov-Smirnov test and of the homocedasticity by the Bartlett test [22].

## Results

### Knowledge related to reproduction

The recorded size and ages that the fish begin to reproduce at were not different for either city-based fishermen or riverine fishermen (P > 0,05). The first maturation length and age was 20 ± 1.5 cm CT and 1,9 ± 0.6 year^-1^, respectively. The length of 100% sexual maturity was 27 ± 2,4 cm CT, without significant difference among fishermen (P > 0,05). The age of 100% sexual maturity was significantly different between fishermen (P < 0,05), where the city-based fishermen indicated full maturation between 5 ± 2,5 years old and the riverine ones registered 2,3 ± 0,7 years old.

**Figure 1:**
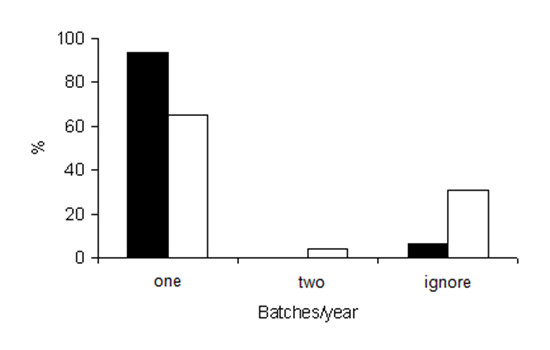
**Spawning frequency of jaraquis per year according to city-based (black bars) and riverine (white bars) fishermen**.

The number of spawning seasons were estimated differently among city-based and riverine fishermen (P < 0,05). It was registered that 93,5% of the city-based fishermen affirmed knowing that the species only spawns once a year, while of the riverine fishermen, just 65% affirmed that the fish spawn only once a year, and 31% said that they would not know how to answer (Figure 1).

More common were declarations that jaraquis spawn in white water rivers during the beginning of the flood season. No parental care was recorded and the declarations about fecundity usually ranged from a thousand to 50 thousand eggs per batch according to fishermen. However, the fecundity information was very diversified (350 to 1 million eggs according to city-based fishermen and 6,5 thousand to 70 thousand eggs according to riverine fishermen). The difficulties encountered by fishermen for this type of information were so great that just 17% of the riverine and 51% of the city-based fishermen gave an answer on the subject. The declared information is presented concisely along with data of the scientific literature in Table 1 to proceed for further discussion.

**Table 1:** Cognitive map about jaraquis reproduction according to central Amazon fishermen and the scientific literature.

ITEM	CITAÇÕES DOS PESCADORES	CITAÇÕES CIENTÍFICAS
Length of first maturation	"The fish begins to reproduce with 20 cm and 2 years old"	Jaraqui-escama-fina: L_50_: 24-25 cm FL [47] Age L_50_: 2,0-2,2 years [10] L_50_:24,8 cm TL [42]
	"The jaraqui (reproducer) length is 27 cm (CT) with about 5 years old"	Jaraqui-escama-grossa: L_50_:22,3 cm SL/2,3 years [9] Age L_50_: 1,9-2,3 years [10] L_50_:26,4 cm TL [42] Jaraquis: Age L_50_: 2,0 years [41]
		Jaraquis: Age L_50_: 2,0 years [41]

Spawning season	"Jaraqui produces youths during the floods "	Jaraqui-escama-fina: Dec-Jan [38] Jaraqui-escama-grossa: Jan-Mar [42] Jaraquis: Dec-Mar/beginning of the flood^1 ^[41,47]; Nov-Mar [9]

Spawning grounds	" Jaraqui spawns in white waters"	Spawn in the confluence of black and white waters [41,42]

Parental care	"Jaraqui do not take care"	Parental care not recorded [41-43,47,50]

Spawning type	"Jaraqui spawn once per year"	Total spawners [41,42,50]

Fecundity	"The fish has many eggs, thousands"	Jaraqui-escama-fina: 45-105 thousand [57]

### Knowledge related to migratory activities

**Figure 2:**
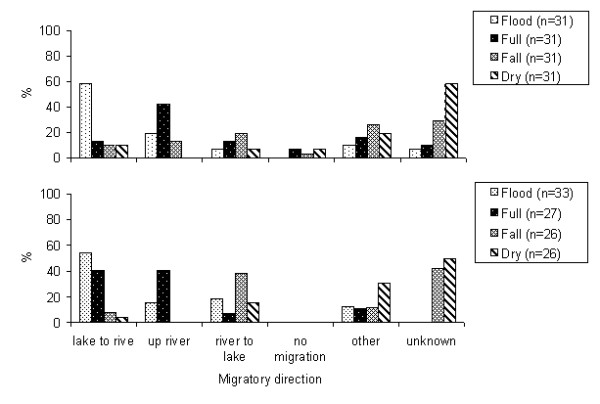
**Main migratory activity of jaraqui along an year according to city-based (above) and riverine (below) fishermen, discriminating hydrological cycles: flood, full, fall and dry season**.

Both city-based fishermen and the riverine fishermen (P > 0,05) stated that during the beginning of the flood, the jaraquis were leaving the lakes and small creeks to spawn in the main river (Figure 2). The occurrence of another large scale migration, mainly the dispersion migration at the end of the flood and during the full season was mentioned by about 40% of the professional and riverine fishermen. Lateral migrations are more often mentioned for riverine than for city-based fishermen, and these were the only declarations indicating that jaraquis do not migrate laterally.

### Knowledge related to trophic ecology

**Figure 3:**
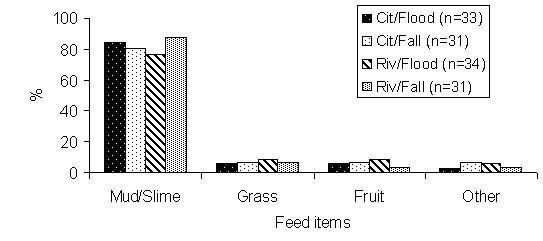
**Feeding preferences of jaraqui according to city-based and riverine fishermen, discriminating the flood and fall seasons**.

According to the fishermen, the jaraquis feeds preferentially in mud or slime, indicating the lake as a feeding environment during the flood and drain water seasons. They also stated that there is no indiscriminate alteration in the items of the alimentary diet if the waters are rising or falling. Some fishermen emphasized the decrease in food availability during the period of drought, but without significant difference (P > 0,05) among the answers of the types of fishermen (Figure 3).

**Figure 4:**
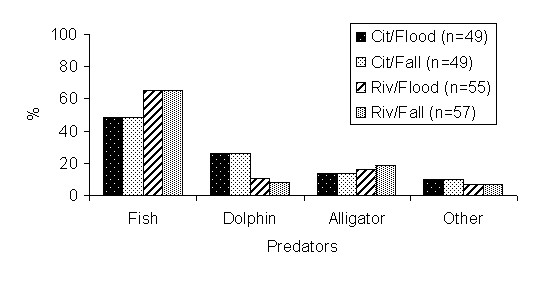
**Main predators of jaraqui according to city-based and riverine fishermen, discriminating the flood and fall seasons**.

According to fishermen (without significant differences P > 0,05), the main jaraquis predators are other fish, for example, catfish: pirarara (*Phractocephalus hemiliopterus*), piraíba (*Brachyplatystoma filamentosum*), dourada (*Brachyplatystoma rousseauxii*), pacamum (*Zungaro zungaro*) and surubim (*Pseudoplatystoma *spp.). Piranha (Serrasalminae), tucunaré (*Cichla *spp.) and pirarucu (*Arapaima gigas*) are also examples of other fish that feed on jaraquis during all seasons (Figure 4).

### Knowledge related to age and growth

The fishermen stated that the average of size of a one-year-old jaraqui is around 15 cm in total length (TL). For two-years-old fish, the average length measured was 20 cm TL and for 3 years-old-fish the estimation reached 23 cm TL (Table 2), without significant differences among the types of fishermen (P > 0,05).

**Table 2:** Jaraqui growth according central Amazon fishermen and comparing to that described by the scientific literature.

Age	Fishermen	Literature*
1	15,1 ± 2,8 cm CT	14,6 cm SL
2	20,3 ± 2,7 cm CT	22,8 cm SL
3	22,7 ± 5,7 cm CT	25,2 cm SL
Larger/older fish	-	39-41 cm SL

* Jaraqui-escama-grossa [9]

### Knowledge related to mortality and fish recruitment

**Figure 5:**
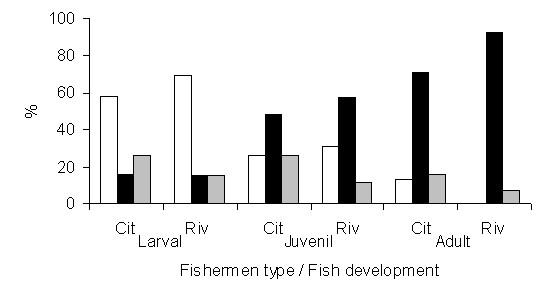
**Natural mortality (white bars), fishery mortality (white bars) and ignored causes of mortality (gray bars) of jaraqui for larval, juvenile and adult development phases, according to city-based and riverine fishermen**.

According to the fishermen, mortality by natural causes is the main *causa mortis *of the jaraquis during the larval and juvenile phases, indicating a feeding preference for other fish as the main factor (Figure 5). For the adult phase, the fishermen indicated fishing as the main cause of mortality (without differences between types; P > 0.05).

The fishery recruitment size of the jaraquis according to both types of fishermen (P > 0,05) was 20,1 ± 2,3 cm TL.

## Discussion

The economic and social importance of fishing resources is commonly highlighted in scientific or socioeconomic projects, even though there are few circumstances where there is effective management that allows sustainable and productive use of these resources [23], generating the example of the Tragedy of the Commons [24,25].

Considering the importance of fishing resources, why is management failure so common? Several elements have been discussed concerning different aspects of these failures (e.g. [26-29]), and delays associated to centralized management arise as an important contributing factor. Co-management is an alternative proposed by several authors (e.g. [30-33]) and is being developed in several fisheries (e.g. [31,34,35]). However, how is it that managers and resource users obey pre-established norms that they do not understand and that sometimes go against common sense? A typical example of this happens in developing fisheries, where an accumulated stock generates growing yields in response to increases in fishing effort (e.g. [36]). In these circumstances, the political environment does not allow managers to take hard decisions. Thus, it is necessary to understand the users' knowledge and associate it to the technical and scientific knowledge in a comprehensible way in order to justify stakeholders' decisions.

Studies of Brazilian ethnoichthyologist pioneers have already shown that fishermen possess knowledge and carryout practices related to the structure and function of the ecosystems they inhabit, which enables them to be powerful partners in natural resource management [15,16,37,38].

The jaraquis are the principal fishing resource in the Central Amazon, requiring specific management strategies, although they are exploited in a multi-specific system that fishes migrating Characiforms using purse seine nets [23]. There are numerous difficulties in the execution of command-control strategies in the region, which makes co-management an alternative to be developed, requiring that users and governmental managers share knowledge to generate a common knowledge to be used in the management.

In order to find common bioecological knowledge between fishermen and scientific literature, it is necessary to compare them without the objective of evaluating the quality of one against the other, but merely to find common characteristics close enough to facilitate dialogue and the comprehension to share decisions. Hence, this research confirms the existence of kindred knowledge in some areas but strong differences in others.

As regards knowledge related to reproductive behavior, we observed that the information is usually related to the scientific knowledge. However, for fish of larger size and therefore of greater age similarities were fewer.

In the view of the fishermen, the size of sexual maturity was similar among city-based and riverine people, however when comparing its results with the scientific knowledge published, the concept of the size of sexual maturity needed to be standardized. In fishing research, the size of sexual maturation is the length in which 50% of a population are capable of reproduction [39,40], but when asked about the size of sexual maturation or "At which size does it begin to reproduce?", fishermen indicated the smallest fishes that had developed gonads. This may be an answer to the question "At which size do all fishes mature?", which indicates only the sizes of mature individuals. Considering the symmetry of the logistic curve [39], an average of 23,5 cm TL was estimated, which is compatible with sizes registered in the scientific literature. The same occurred with the age of first sexual maturity, estimated to be around 2 years.

The reproductive behavior has common features indicating that the jaraqui begins its reproductive period in the flood season, in downstream shoals, spawning in the confluence of the white and black water [41,42]. Vieira *et al. *[43] identified the following spawning environments for the jaraqui: black water creek mouth, black river mouth and small lake creeks (a thin water connection between black water lakes and white water rivers).

Vazzoler *et al. *[42] and Ribeiro and Petrere [41] do not indicate that there is any parental care, which was compatible with the answers of the fishermen and with the typical behavior of Characiforms in the Amazon region [44].

Only few fishermen (just 51% of the professional and 17% of the riverine fishermen) were confident to confirm anything about the fecundity. Their answers were very diverse, indicating the difficulty in observing values in an appropriate magnitude. The fecundity is a variable usually correlated with the length of the female based on a potential model [39]. This increases the variability of the fecundity for any species and makes it difficult for fishermen to record. Even in scientific research there are few recordings for the species that reach a magnitude of thousands of eggs. This magnitude increases the difficulties in cultural registration, which may be different for k-strategic species, with few and easily countable eggs.

In relation to patterns of migration, riverine and city-based fishermen pointed out that the jaraquis were leaving the lakes during the flood season. In the scientific literature we found evidence that jaraquis form shoals that perform longitudinal migrations of the order of 1000 to 1300 km every year [41,45], with a maximum displacement of around 300 km in white water rivers for reproduction, feeding and dispersion. In a general way, the migration of this species is considered very complex, being observed and described in a general model by Goulding [46], and detailed later on by Ribeiro [47]. This description of the migratory circuit was also confirmed by Vazzoler and Amadio [45] and Vieira *et al. *[48]. The fat-fish migration phase described by Ribeiro [47] is particularly important for the dispersion of the jaraquis to other tributaries of the Solimões-Amazonas system and happens in the middle of the full-flood seasons (from March to June). This was also registered as important by fishermen. Lateral migrations are very important type of behavior in the ecology of the Characiformes [49], but it was confirmed more frequently by riverine fishermen, which is natural considering the limited mobility of these people who therefore had more scope to examine aspects of the local dynamics.

The information provided by the fishermen about trophic ecology was similar to that found in the scientific literature, where Ribeiro [47], Vazzoler *et al. *[42] and Barthem and Fabré [50] observed that the jaraqui presents detritivorous habits. More specifically, Ribeiro [47] registered some of the items included in its diet, such as periphyton on the trees, submerged leaves, diatoms, sponge spicules, mushrooms and bacteria, mainly in the full season when the fish are dispersed in the flooded forests. During this period the mud or slime indicated by the fishermen is compatible with scientific information but with fewer details. Fishermen also indicated lakes and flooded areas as being important for its feeding in the flood and fall seasons.

On the other hand, the fishermen of the area pointed out that the jaraquis is predated by alligators, dolphins and other piscivorous fish, particularly catfish of several species. This corresponds to Ribeiro's research [47], which mentions the great catfish, as the piraíba (*Brachyplatystoma filamentosum*) in the white water rivers, as the main predators of the species along with the dolphins, not to mention the alligators.

In growth analysis, significant divergences were expected, due to the fact that information on age is not available for researchers or fishermen, both facing difficulties in the identification of modal length groups. The count and measurement of age rings in rigid structures also present specific difficulties for scientists [51,52], but have not been used by fishermen. Such differences contributed to the high dispersion between the values mentioned by fishermen and these found in the scientific literature.

Regarding mortality, fishermen have made clear that for larvae and juveniles, predators are the key factor, but also that fishing is the main factor for the adults. The latter corresponds to the high rate of fishing exploitation observed for the jaraquis in research in the area [8,10] and the former to the findings of Bayley [53]. However, the fishermen's findings were very superficial and without details particularly for young fish, since these kinds of observation are not useful to them. Hence, fishermen's mortality estimates do not correspond to the instantaneous coefficient of mortality in fishing biology assessments, but rather to the causes of mortality and the variation in intensity.

The mean size of the first catch recorded in the literature indicated that commercial fishing has been concentrated on one year old jaraquis [8]. This fishing recruitment corresponds to jaraquis-escama-grossa usually from 24 to 27 cm CF and jaraquis-escama-fina still smaller (21-22 cm CF), values which are superior to the total length of 20.1 cm indicated by the fishermen. These differences of perception could indicate that the catch is being made of individuals smaller than those observed in the landing harbors, due to the fishermen selecting larger fishes.

Evaluating all the information comparatively, compatibilities in factors were found related to the use of the resource that make both fishermen types into good observers. However, if certain phenomena do not directly affect the interests of the fishermen, these tend to be ignored. The professionals seem to be more efficient in gathering general information, while the riverine fishermen were more efficient in gathering information that required local observations.

Compatibility of knowledge is, however, not enough; it is also necessary to develop a culture of dialogue between stakeholders and users in the establishment of more efficient and sustainable strategies. User knowledge has been gathered by researchers and may contribute as relevant information to government management plans [[33]], particularly for resources with scarce or non-existent data (e.g. [54-56]). On the other hand, ethnoecological knowledge can also be the basis for the design of management plans, as has been done by some Amazon communities [17,18], and are the informational basis for fishing agreements recognized by government environmental authorities in Brazil (IBAMA normative instruction 29/2002). Ethnoecological research may help the dialogue between fishermen, researchers and stakeholders, increasing willingness and cooperation to find effective solutions to optimal management of fish resource but also of the environment.

The present paper suggests some themes that can be used to introduce affinities in order to facilitate this dialogue but this only represents one initiative among many others necessary to effectively put in place a co-management regime. Success in this task can change a centralized decision making culture and encourage collaboration in the productive and sustainable use of fishing resources in the region.

## Conclusions

Dialogue between managers and fishermen would develop better if discussions concentrated on information about the reproductive cycle, spawning and nursery grounds, maturity size and feeding preferences of these species. Age and growth as much as mortality were useful information on scientific grounds, but their use in co-management is minimized due to difficulties encountered by fishermen in understanding and appreciating the estimates done. Strategies aimed at developing the dialogue could include short practical courses to encourage fishermen (and also their families) to observe subjects in order to improve their understanding of technical procedures and results and to enable the use of age-related and mortality knowledge. Current thinking suggests concentrating on co-management discussions on the improvement of area and seasonal restrictions.

## Competing interests

The authors declare that they have no competing interests.

## Authors' contributions

The authors of this paper were equally responsible for every aspect of the research, the conclusions, and the writing of the paper. All authors read and approved the final manuscript.
